# Ballistic Behavior of Bioinspired Nacre-like Composites

**DOI:** 10.3390/biomimetics8040341

**Published:** 2023-08-01

**Authors:** Danny G. Chan-Colli, Eliana M. Agaliotis, David Frias-Bastar, Luming Shen, Jose G. Carrillo, Pedro J. Herrera-Franco, Emmanuel A. Flores-Johnson

**Affiliations:** 1 Centro de Investigación Científica de Yucatán, Unidad de Materiales, Calle 43 No. 130 Col. Chuburná de Hidalgo, Mérida 97205, Yucatán, Mexico; danny_gcc16@hotmail.com (D.G.C.-C.); frias_888@hotmail.com (D.F.-B.); jgcb@cicy.mx (J.G.C.); 2 Facultad de Ingeniería, Universidad de Buenos Aires, Av. Las Heras 2214, Buenos Aires C1127AAR, Argentina; eagaliotis@fi.uba.ar; 3 Instituto de Tecnología en Polímeros y Nanotecnología (ITPN), CONICET-Universidad de Buenos Aires, Av. Las Heras 2214, Buenos Aires C1127AAR, Argentina; 4 School of Civil Engineering, The University of Sydney, Sydney, NSW 2006, Australia; luming.shen@sydney.edu.au; 5 Australian Nuclear Science and Technology Organization (ANSTO), Lucas Heights, NSW 2234, Australia; 6 School of Mechanical and Manufacturing Engineering, University of New South Wales (UNSW Sydney), Sydney, NSW 2052, Australia

**Keywords:** bioinspired composite, nacre-like composite, ballistic performance, impact behavior, finite element simulation, cohesive interface, elastoplastic projectile, multilayer plate, layered structure, aluminum alloy

## Abstract

In this paper, the ballistic performance of a multilayered composite inspired by the structural characteristics of nacre is numerically investigated using finite element (FE) simulations. Nacre is a natural composite material found in the shells of some marine mollusks, which has remarkable toughness due to its hierarchical layered structure. The bioinspired nacre-like composites investigated here were made of five wavy aluminum alloy 7075-T651 (AA7075) layers composed of ~1.1-mm thick square tablets bonded together with toughened epoxy resin. Two composite configurations with continuous layers (either wavy or flat) were also studied. The ballistic performance of the composite plates was compared to that of a bulk monolithic AA7075 plate. The ballistic impact was simulated in the 300–600 m/s range using two types of spherical projectiles, i.e., rigid and elastoplastic. The results showed that the nacre plate exhibited improved ballistic performance compared to the bulk plate and the plates with continuous layers. The structural design of the nacre plate improved the ballistic performance by producing a more ductile failure and enabling localized energy absorption via the plastic deformation of the tablets and the globalized energy dissipation due to interface debonding and friction. All the plate configurations exhibited a better ballistic performance when impacted by an elastoplastic projectile compared to a rigid one, which is explained by the projectile plastic deformation absorbing some of the impact energy and the enlarged contact area between the projectile and the plates producing more energy absorption by the plates.

## 1. Introduction

The demand for lightweight materials with impact-energy absorbing characteristics for ballistic applications is constantly increasing, which poses scientific, design and technological challenges to balance the competing constraints of lightweight on the one hand and mechanical performance on the other hand [1]. In this context, biomimetics has emerged as a powerful area of materials science that involves a process of inspiration from natural materials and structures to produce high-performing synthetic materials to solve engineering problems [2]. The evolution of nature over millions of years has resulted in the development of natural materials and structures that can exhibit high mechanical performance by combining materials with ordinary mechanical properties in a hierarchical manner [3].

Biological materials such as wood, bone and marine shells have a complex hierarchical organization through their intrinsic design from the nanoscale to the macroscale [4]. Several energy-absorbing mechanisms interact when these natural materials deform, resulting in high mechanical performance exhibiting a remarkable combination of stiffness, strength and toughness.

Among these natural materials with outstanding mechanical properties, nacre has raised significant interest as a structural system for materials science and engineering. Nacre, the iridescent material found in mollusk shells, is a biological material that exhibits remarkable mechanical properties due to its complex hierarchical structure, which spans several length scales [5,6]. Nacre is a “brick–mortar” composite structure, in which the “bricks” are tablets made of aragonite (a brittle mineral) and the “mortar” is a soft organic biopolymer that “glues” the tablets together [7]. Notwithstanding that nacre is 95% aragonite, it has a toughness ~3000 times higher than aragonite [8]. This remarkable mechanical behavior is attributed to the “brick–mortar” structural arrangement, the waviness of the tablets, and the interfacial behavior between the tablets [9,10].

While extensive experimental work has been focused on understanding the microstructure and mechanical behavior of the natural nacre material [7,11,12], investigation of the mechanical performance of nacre-like synthetic composites at the macroscale (millimeter scale designs) is still ongoing [13,14]. Barthelat and Zhu [15] developed a millimeter-size prototype of poly-methyl-methacrylate tablets based on the nacre structure. They showed the effect of the tablets’ waviness on the toughness of the nacre-like composite. Ko et al. [16] investigated the low-velocity impact performance of 3D-printed polymer nacre-like composites and found that the nacre-like composites outperformed an equivalent monolithic plate under impact loading. Wu et al. [17] also investigated 3D-printed polymer nacreous structures subjected to drop-weight impact loading. They found that the nacreous composites’ impact performance was improved by tuning the interfacial strength between the layers. Yin et al. [18] investigated a nacre-like glass composite subjected to low-velocity impact. They found that the nacre-like glass structure had a higher impact resistance than laminated and tempered glass. Miao et al. [19] performed ballistic impact tests on nacre-like composites fabricated with aluminum alloy and epoxy resin. They found that the nacre-like plates exhibited a better ballistic performance by producing a lower residual velocity of the projectile than their equivalent bulk plates.

Numerical simulations have also been used to understand the impact behavior of nacre-like composites. Knipprath et al. [20] developed a finite element (FE) model of boron carbide ceramic composites and showed that the composites’ ballistic impact response could be improved using a simplified nacre-like structural design promoting crack delocalization. Yang et al. [21] numerically investigated the ballistic behavior of semi-cylindrical nacre-like composite shells of brittle silicon carbide ceramic and aluminum alloy. They found that the nacre-like composites had a higher ballistic limit than the shells made of only aluminum alloy. Gao et al. [22] performed FE simulations to study the impact resistance of nacre-like aluminum alloy composites with Voronoi-shaped tablets and epoxy adhesive. They found that increasing the number of tablet layers and reducing the number of polygons within the same layer improved the global energy absorption and impact performance. Ghazlan et al. [23] developed FE simulations of a nacre-like composite made of silicon carbide and Kevlar layers. They found that the bioinspired panels had better ballistic performance than the monolithic plates. Flores-Johnson et al. [24] investigated the ballistic performance of nacre-like composite plates of aluminum alloy tablets bonded with toughened epoxy resin. They found a performance improvement in the nacre-like plate compared to a bulk aluminum plate of the same thickness.

Based on the literature mentioned above, several studies have investigated the ballistic impact performance of bioinspired composites made of aluminum alloys; however, most of these investigations were performed using rigid or hardened steel projectiles, which exhibited little or no deformation. However, our previous experimental work [19] showed that projectiles made of non-hardened steel, such as stainless steel, can plastically deform during impact and modify the penetration process of the bioinspired structure. In this paper, we further investigate the projectile’s plastic deformation effect on the impact performance of nacre-like composites, which is essential to better understand the ballistic behavior of these bioinspired structures. Hence, this paper presents FE simulations of five-layer nacre-like aluminum alloy composites subjected to ballistic impact by either an elastoplastic or a rigid projectile with impact velocities in the 300–600 m/s range. The nacre-like composite design, based on our previous numerical work on bioinspired composites [24], consists of nacre-like plates with five layers made of ~1.1 mm thick aluminum alloy 7075-T651 (AA7075) composed of square tablets which are bonded together with a toughened epoxy resin. Furthermore, we present the ballistic performance of two additional composite configurations with five continuous, either wavy or flat, layers. Finally, we compare the ballistic performance of the bioinspired composites with that of a bulk monolithic AA7075 plate of the same thickness when impacted by the elastoplastic and rigid projectiles.

## 2. Methods

### 2.1. Nacre-like Composite Plate Configurations

**Figure 1:**
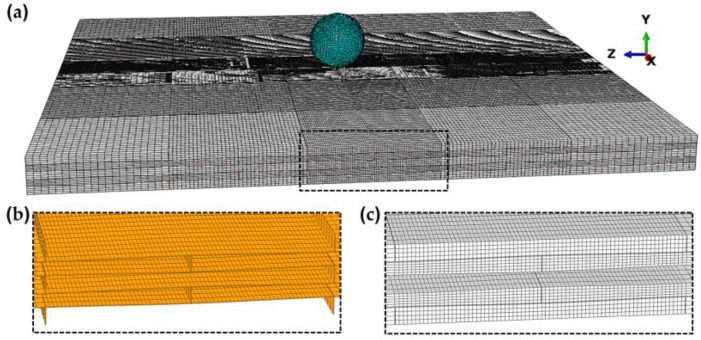
(**a**) FE mesh of the nacre-like composite plate; (**b**) close-up view of the cohesive element layers; and (**c**) close-up view of the brick element layers (the middle brick element layer is not displayed to show the interior).

Composite plates with dimensions of 100 mm × 100 mm and a thickness of 5.4 mm were impacted by a steel spherical projectile (Figure 1a) with a diameter of 10 mm, a mass of 4.4 g and initial impact velocities in the 300–600 m/s range. Three different composite plate configurations were investigated. First, the nacre-like composite plate (nacre plate) inspired by the structural characteristics of the nacre material was simulated, which included wavy layers, layers composed of several tablets, and cohesive interfacial behavior between the layers and individual tablets (Figure 1b,c).

**Figure 2:**
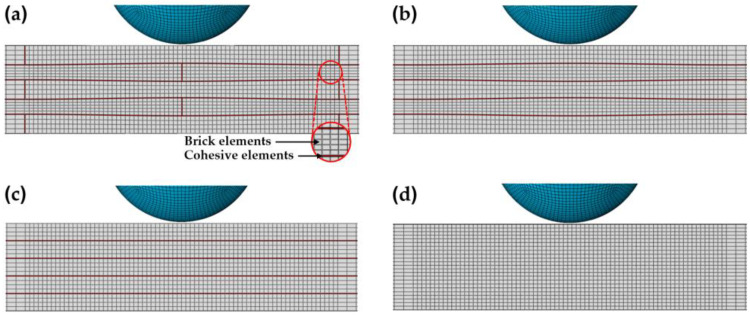
Cross section of the FE mesh of the various plate configurations: (**a**) nacre plate; (**b**) wavy plate; (**c**) flat plate; and (**d**) bulk plate.

The nacre plate consisted of five wavy layers of aluminum alloy 7075-T651 (AA7075) with a thickness of ~1.1 mm, giving a total thickness of 5.4 mm (Figure 2a). Each layer of the nacre plate consisted of twenty-five 20 mm × 20 mm square tablets. In addition, each tablet was displaced with respect to its adjacent upper and/or lower neighboring tablet such that individual tablets overlapped 1/4 of the surface area (Figure 2a) [24]. The waviness of the layers was created using a sinusoidal function with a wavelength of 20 mm and an amplitude of 0.1 mm (Figure 2a). Second, a composite plate with continuous wavy layers (wavy plate) was modelled (Figure 2b). For this configuration, five continuous wavy layers of AA7075 with a thickness of ~1.1 mm were employed. Third, a composite plate with flat continuous layers of AA7075 (flat plate) was modeled (Figure 2c). For the three configurations mentioned above, the epoxy resin Betamate 1044 was employed to model the interface between the AA7075 layers (and between the tablets of each layer in the nacre plate configuration). In addition, a monolithic plate (bulk plate) made of AA7075 (Figure 2d) with an equivalent thickness was also simulated to compare its performance with that of the composite plate configurations.

### 2.2. Finite Element (FE) Model

The ballistic behavior of the various plate configurations was investigated using FE simulations performed with the software Abaqus/Explicit (Version 2016, Dassault Systèmes Simulia Corp., Providence, RI, USA) [25]. The solid AA7075 layers of the composite and bulk plates were meshed using linear brick (hexahedral) elements with reduced integration (C3D8R) and enhanced hourglass control (Figure 1c). The epoxy resin between the layers and tablets was modelled using three-dimensional cohesive elements (COH3D8) with a thickness of 0.05 mm (Figure 1b). The adjacent elements between the solid and cohesive regions shared nodes. The spherical projectile was also modeled using brick elements. Two different cases were simulated for the projectile mechanical behavior, i.e., the projectile was modeled with an elastoplastic behavior and as a rigid body. The mesh was refined at the central region of the plates (impact region) with an average element size of 0.27 × 0.27 × 0.27 mm^3^ (Figure 1a). This element size was selected based on a mesh sensitivity analysis presented in our previous work [24]: six different element sizes between 0.21 × 0.21 × 0.21 mm^3^ and 1 × 1 × 1 mm^3^ were used in a numerical model of a 20-mm thick AA7075 plate impacted by blunt and ogival projectiles. The results showed that the element size of 0.27 × 0.27 × 0.27 mm^3^ was sufficient for convergence and computational efficiency [24]. The plates were fully clamped at all the edge boundaries. Finally, a penalty friction formulation was used for the tangential contact between the projectile and the plates and between the delaminated layers; a coefficient of friction of 0.2 was employed.

### 2.3. Materials

#### 2.3.1. Johnson–Cook Plasticity Model and Fracture Criterion

The plastic behavior of the AA7075 was simulated using the Johnson–Cook (JC) plasticity model [26]. The JC model is an empirical model that can be expressed as:

(1) $$\sigma_{eq}=(A+B\varepsilon_{eq}^{n})\left(1+Cln\frac{{\dot{\varepsilon }}_{eq}}{{\dot{\varepsilon }}_{0}}\right)(1-{\hat{T}}^{m})$$

where *A*, *B*, *n*, *C* and *m* are material constants, $\sigma_{eq}$ is the equivalent yield stress, $\varepsilon_{eq}$ is the equivalent plastic strain, ${\dot{\varepsilon }}_{eq}$ is the equivalent plastic strain rate, and ${\dot{\varepsilon }}_{0}$ is a user-defined reference strain rate. $\hat{T}$ is the homologous temperature defined as $\hat{T}=(T-T_{r})/(T_{m}-T_{r})$, where T is the absolute temperature, $T_{r}$ is the room temperature, and $T_{m}$ is the melting temperature. The JC plasticity model was used in conjunction with the Johnson–Cook (JC) fracture criterion [27] to model the damage accumulation. The JC damage criterion is based on the accumulation of the equivalent plastic strain at element integration points. Failure occurs when the damage parameter ω exceeds 1; ω is defined as [25]:

(2) $$\omega =\sum \frac{∆\varepsilon_{eq}}{\varepsilon_{f}^{JC}}$$

where $∆\varepsilon_{eq}$ is an increment of the equivalent plastic strain, and $\varepsilon_{f}^{JC}$ is the JC equivalent fracture strain [25,27] defined as:

(3) $$\varepsilon_{f}^{JC}=\left[\left(D_{1}+D_{2}\exp\left({D_{3}\sigma }^{*}\right)\right)\right]\left(1+D_{4}ln\frac{{\dot{\varepsilon }}_{eq}}{{\dot{\varepsilon }}_{0}}\right)(1+D_{5}\hat{T})$$

where $D_{1},\ldots ,D_{5}$ are material constants and $\sigma^{*}$ is the stress triaxiality. In Equation (2), the summation is performed for each element integration point over all the increments in the simulation; when ω ≥ 1, failure occurs.

The material properties and JC plasticity model parameters used for the AA7075 of the composite plates and the projectile’s stainless steel (SS) 316L are depicted in Table 1 and Table 2, respectively. The damage parameters for the JC fracture criterion used for the AA7075 of the composite plates are shown in Table 3. A displacement at failure $u_{pl}^{f}$ = 0.0009 mm was employed for the JC fracture criterion’s damage evolution. It is mentioned that the JC material model and fracture criterion parameters in Table 2 and Table 3 have been experimentally validated against ballistic impact tests [28] in our previous work for the AA7075 [24] and in [29] for the SS 316L (Table 2).

#### 2.3.2. Constitutive Response of the Cohesive Elements

The mechanical response of the epoxy resin used for the interface between the layers and tablets of the composite plates was modeled using cohesive elements with a thickness of 0.05 mm (Figure 1b and Figure 2), obeying a traction–separation law [25]. Three-dimensional cohesive elements (COH3D8) were used. Damage in the cohesive elements was assumed to initiate when the following quadratic interaction function involving the normal $t_{n}$ and shear tractions $t_{s}$ and $t_{t}$ reached a value of one [25]:

(4) $${\left(\frac{t_{n}}{t_{n}^{0}}\right)}^{2}+{\left(\frac{t_{s}}{t_{s}^{0}}\right)}^{2}+{\left(\frac{t_{t}}{t_{t}^{0}}\right)}^{2}=1$$

where $t_{n}^{0}$ is the normal strength and $t_{s}^{0}$ and $t_{t}^{0}$ are the shear strengths. For the damage evolution of the cohesive elements, the energy-based mixed-mode Benzeggagh–Kenane (BK) fracture criterion [25,32] was employed; a BK material parameter η = 2 was used in the simulations. Table 4 shows the material parameters of the epoxy resin used for the cohesive elements.

## 3. Results and Discussion

### 3.1. Ballistic Performance of the Plate Configurations

**Figure 3:**
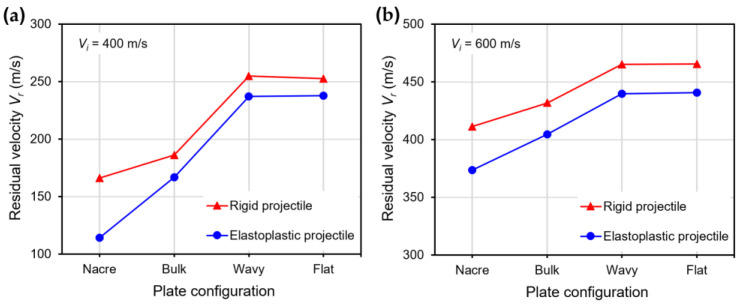
Residual velocities for various plate configurations using rigid and elastoplastic projectiles and impact velocities of (**a**) $V_{i}$ = 400 m/s; and (**b**) $V_{i}$ = 600 m/s.

Figure 3a,b show plots of the residual velocity $V_{r}$ for the impact velocities $V_{i}$ of 400 and 600 m/s, respectively, for all the plate configurations using rigid and elastoplastic projectiles. It can be seen that for both impact velocities, the best-performing plate configuration (exhibiting lower $V_{r}$) was the nacre plate, followed by the bulk plate. In contrast, the wavy and flat plates performed the worst, showing a similar $V_{r}$. For $V_{i}$ = 400 m/s, the nacre plate enabled a 10.8% and 31.4% reduction in the residual velocity compared to the bulk plate for the rigid and elastoplastic projectiles, respectively. Furthermore, the nacre plate produced a 34.2% and 51.8% residual velocity reduction for the rigid and elastoplastic projectiles, respectively, compared to the wavy and flat plates.

**Figure 4:**
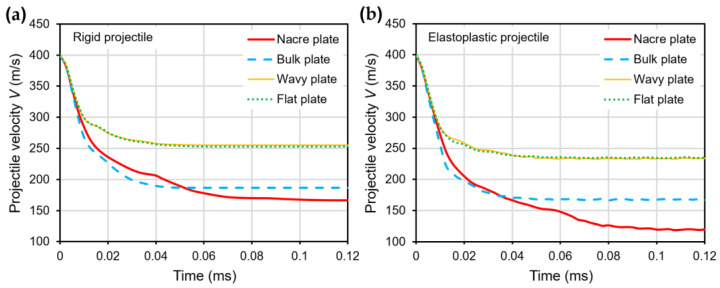
Projectile velocity–time curves for various plate configurations using (**a**) rigid and (**b**) elastoplastic projectiles with $V_{i}\;$= 400 m/s.

**Figure 5:**
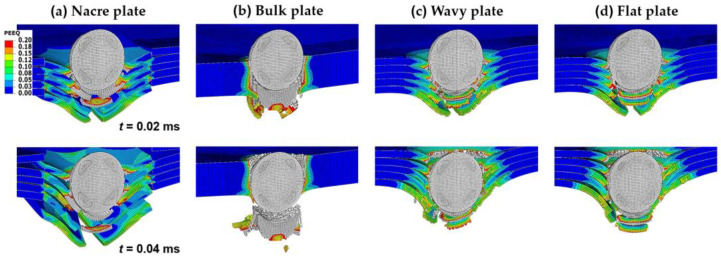
Contour plots of the equivalent plastic strain (PEEQ) at *t* = 0.02 ms and *t* = 0.04 ms of various plate configurations impacted by a rigid projectile with an initial velocity of 400 m/s: (**a**) nacre plate; (**b**) bulk plate; (**c**) wavy plate; and (**d**) flat plate. Areas with high plastic strain levels larger than 0.2 are indicated in gray.

**Figure 6:**
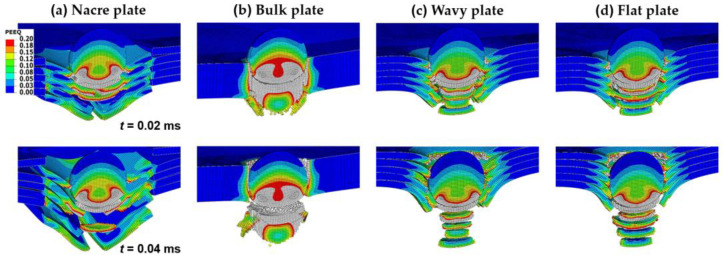
Contour plots of the equivalent plastic strain (PEEQ) at *t* = 0.02 ms and *t* = 0.04 ms of various plate configurations impacted by an elastoplastic projectile with an initial velocity of 400 m/s: (**a**) nacre plate; (**b**) bulk plate; (**c**) wavy plate; and (**d**) flat plate. Areas with high plastic strain levels larger than 0.2 are indicated in gray.

Figure 4a,b show the velocity V history of the rigid and elastoplastic projectiles, respectively, for all the plate configurations impacted with $V_{i}$ = 400 m/s. At time *t* = 0.02 ms, when the projectile has penetrated the plates halfway (Figure 5 and Figure 6), the V is already much lower for the nacre and bulk plates than for the wavy and flat plates for both the rigid and elastoplastic projectiles. It can also be seen that at *t* = 0.02 ms, the V is slightly higher for the nacre plate compared to the bulk plate; however, at *t* = 0.04 ms, when the projectile has fully penetrated the plate (Figure 5 and Figure 6), the V is lower for the nacre plate, which indicates a better ballistic performance.

The observed better ballistic performance (lower projectile velocity at full penetration) of the nacre plate is explained by the reduction in the bending stiffness in the other plates due to the brittle failure at the back of the plate compared to the ductile failure of the nacre plate. This behavior can be observed better in Figure 5 and Figure 6, which show contour plots of the equivalent plastic strain (PEEQ) of the various plate configurations, at *t* = 0.02 and 0.04 ms, impacted by rigid and elastoplastic projectiles, respectively. The better performance of the nacre plate is produced by the bioinspired structural design, which enables both localized energy absorption via the plastic deformation of the tablets and globalized energy dissipation due to interface debonding and friction. It is noted that the interaction between the surfaces is further augmented due to the tablet waviness. Figure 5b and Figure 6b show that the bulk plate fails by brittle fracture and fragmentation, resulting in a localized plastic deformation compared to the larger area of plastic deformation observed in the nacre plate (Figure 5a and Figure 6a).

**Figure 7:**
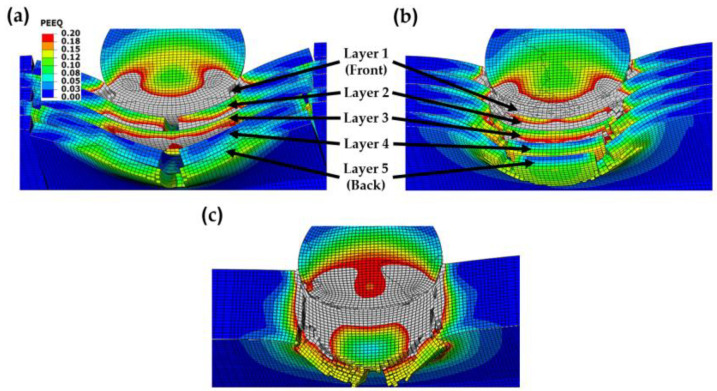
Contour plots of the equivalent plastic strain (PEEQ) at *t* = 0.015 of the (**a**) nacre plate; (**b**) flat plate; and (**c**) bulk plate impacted by an elastoplastic projectile with an initial velocity of 400 m/s. Areas with high plastic strain levels larger than 0.2 are indicated in gray.

The lower impact performance of the wavy (Figure 5c and Figure 6c) and flat (Figure 5d and Figure 6d) plates is explained by the more brittle behavior exhibited by these configurations compared to the nacre plate (Figure 5a and Figure 6a). This behavior can be better observed in Figure 7a,b, in which the nacre and flat plates are shown at *t* = 0.015 ms, respectively, when impacted by an elastoplastic projectile with an initial velocity of 400 m/s. It can be seen that for the wavy plate (Figure 7b), all five layers have failed in a brittle manner, primarily via shear failure. In contrast, for the nacre plate (Figure 7a), layer 1 (front face) failed via shear failure and delamination, layers 2 and 4 via delamination in the middle part, and layers 3 and 5 via delamination between adjacent tablets; for layer 5 (back face), tensile failure is also observed in the middle section. These observations confirm that the improved impact performance of the bioinspired composite is due to the various interacting energy-absorbing mechanisms enabled by the bioinspired design, which are not exhibited by the other plates. Figure 7c shows the bulk plate’s shear failure (plugging), which is the primary failure mechanism observed for this plate configuration. These observations (Figure 6b and Figure 7c) agree with the bulk plate observations during impact in our previous experimental work, in which shear plugging and severe fragmentation were observed [19]. In contrast, for the nacre plate, small fragments and ejected delaminated tablets were observed experimentally [19], as is captured in our numerical simulations (Figure 6a).

### 3.2. Effect of the Elastoplastic Behavior of the Projectile on the Ballistic Performance

**Figure 8:**
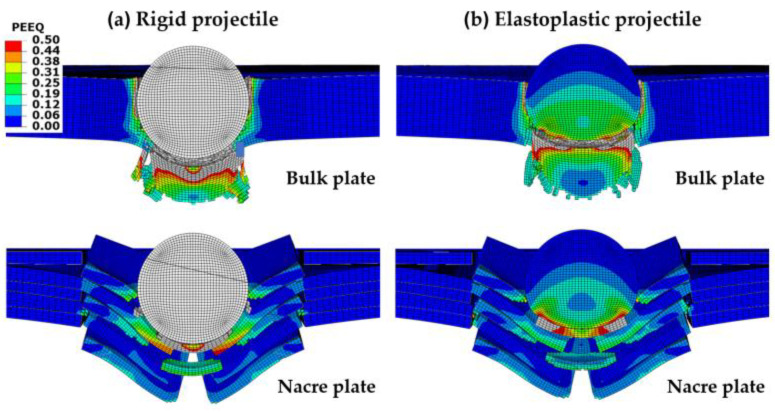
Contour plots of the equivalent plastic strain (PEEQ) at *t* = 0.03 ms of the nacre and bulk plates impacted by projectiles with an initial velocity of 400 m/s: (**a**) rigid projectile; and (**b**) elastoplastic projectile. Areas with high plastic strain levels larger than 0.5 are indicated in gray.

**Figure 9:**
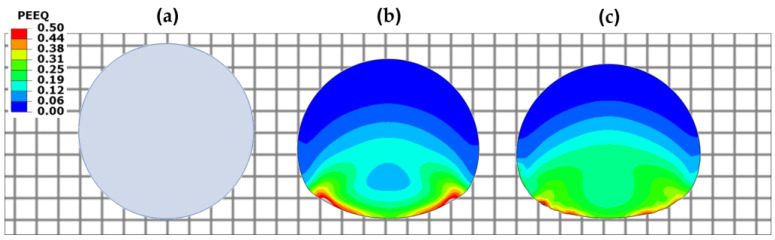
Projectiles impacting the composite plates with an initial velocity of 400 m/s at *t* = 0.06 ms: (**a**) rigid projectile; (**b**) elastoplastic projectile impacting the nacre plate; and (**c**) elastoplastic projectile impacting the bulk plate. Areas with high plastic strain levels larger than 0.5 are indicated in gray.

Figure 3 and Figure 4 showed that the ballistic performance (lower residual velocities) of all the plate configurations was better when impacted by an elastoplastic projectile compared to a rigid one. This observation can be explained by the fact that when the elastoplastic projectile with low strength is used, it deforms plastically (Figure 6), absorbing some of the impact energy. Furthermore, the contact area between the projectile and the plate is enlarged due to the projectile’s plastic deformation, enabling more energy absorption by the plate compared to when impacted by the rigid projectile (Figure 8). Moreover, the elastoplastic projectile impacting the bulk plate exhibits larger plastic deformation compared to the projectile impacting the nacre plate (Figure 8). This behavior is better observed in Figure 9, in which the shape of the rigid projectile (Figure 9a), the projectile impacting the nacre plate (Figure 9b) and the projectile impacting the bulk plate (Figure 9c) are compared at *t* = 0.06 ms when the projectile has penetrated the plates. It is clear that the projectile penetrating the bulk plate exhibited larger equivalent plastic strain and had deformed more than the projectile penetrating the nacre plate. These observations are in accordance with the post-test examination of the stainless steel projectiles used in our previous experimental work, which also exhibited larger plastic deformation when impacting the bulk plate compared to the nacre plate [19]. It is noted that a direct comparison of the numerical results presented here with our experimental results in [19] could not be performed since the cohesive element parameters of the epoxy adhesive 3M Scotch-Weld TM 2216 B/A and the material properties of the stainless steel used in the experimental work were not available for the FE modelling presented here.

### 3.3. Results Analysis

**Figure 10:**
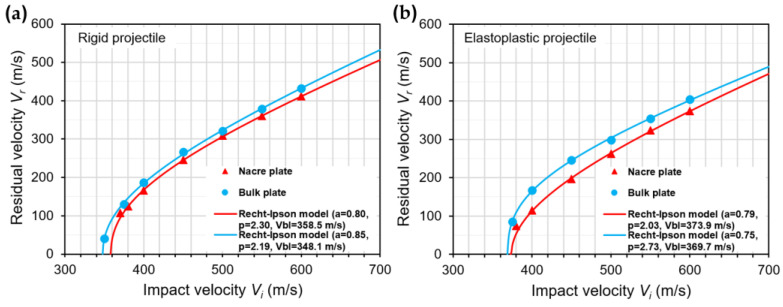
Predicted projectile residual velocity versus impact velocity for the nacre and bulk plates impacted by: (**a**) rigid projectile; and (**b**) elastoplastic projectile. The solid lines represent fits to the predicted data of the Recht–Ipson model.

Figure 10a,b show the FE predictions of the residual velocity versus the impact velocity for the nacre and bulk plates (the two best-performing plate configurations), respectively, when impacted by rigid and elastoplastic projectiles. The solid lines represent fits to the numerical data of the Recht–Ipson model [34] employed to predict the projectile residual velocity $V_{r}$ as follows:

(5) $$V_{r}=a{\left(V_{i}^{p}-V_{bl}^{p}\right)}^{1/p}$$

where a and p are empirical constants used to best fit the data, $V_{i}$ is the initial impact velocity, and $V_{bl}$ is the ballistic limit. The constant a was assumed to be less than 1, considering that fragmentation and plugging were observed during the penetration process of the nacre and bulk plates, respectively (Figure 5 and Figure 6) [35]. The method of least squares was used to obtain the best-fit values of a, p and $V_{bl}$ [36], which are shown in Figure 10. It can be seen in Figure 10 that the trend of the better ballistic performance exhibited by the nacre plate is observed for all the impact velocities, in particular for the elastoplastic projectile with $V_{i}$ > 400 m/s. However, the difference in the residual velocities becomes small for $V_{i}$ < 400 m/s for the elastoplastic projectile, which is explained by the increase in the bending resistance of the bulk plate at low impact velocities [24].

**Figure 11:**
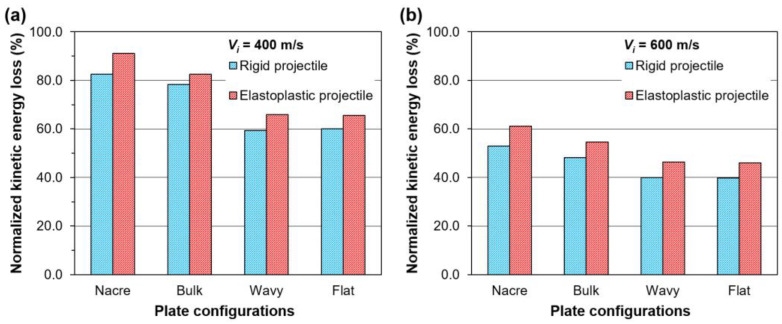
Normalized kinetic energy loss $∆K_{p}$ of the rigid and elastoplastic projectiles after impacting various plate configurations with (**a**) $V_{i}\;$ = 400 m/s; and (**b**) $V_{i}\;$ = 600 m/s.

Figure 11a,b show the normalized kinetic energy loss of the rigid and elastoplastic projectiles after impacting the various plate configurations with impact velocities $V_{i}$ of 400 m/s and 600 m/s, respectively. The normalized kinetic energy loss of the projectile $∆K_{p}/K_{p\_i}$ was calculated as a percentage through the normalization of the kinetic energy loss of the projectile $∆K_{p}$ by the initial kinetic energy of the projectile $K_{p\_i}$. The kinetic energy loss was calculated as $∆K_{p}$ = $K_{p\_i}-K_{p\_r}$, where $K_{p\_i}=\frac{1}{2}\left(M{V_{i}}^{2}\right)$ and $K_{p\_r}=\frac{1}{2}(M{V_{r}}^{2})$; M is the projectile’s mass. It can be seen in Figure 11 that the $∆K_{p}$ of the projectile is higher when impacting the nacre plate compared to the other plate configurations in all cases. For the rigid projectiles, $∆K_{p}$ represents the kinetic energy absorbed by the plate, which is transformed into kinetic energy of the plate and dissipated through the plastic deformation of the aluminum alloy layers, frictional contact and debonding in the case of the bioinspired composites. In the case of the elastoplastic projectiles, $∆K_{p}$ includes the mechanisms of energy absorption mentioned above, and in addition, consists of the projectile’s plastic deformation. For the rigid projectile impacting the nacre plate at 400 m/s (Figure 11a), the $∆K_{p}$ was 5.5% higher than when impacting the bulk plate and ~38% higher than when impacting the wavy and flat plates. For the elastoplastic projectile impacting the nacre plate at 400 m/s, the $∆K_{p}$ was 10.4% higher than when impacting the bulk plate and ~39% higher than when impacting the wavy and flat plates. The improved energy absorption of the nacre plate compared to the bulk plate when impacted with the elastoplastic projectile could be explained by the enlarged contact areas between the projectile and the nacre plate, enabling more energy absorption by the tablets and interfaces further away from the impact zone. For $V_{i}$ = 600 m/s, similar trends in the $∆K_{p}$ were observed for both the rigid and elastoplastic projectiles (Figure 11b).

### 3.4. Discussion

The numerical results presented here have shown that the nacre-like AA7075 composite plates with structural characteristics inspired by the nacre biological material exhibited an improved ballistic performance compared to their equivalent bulk plates. These bioinspired structures could potentially be used as lightweight, protective structures for energy absorption in ballistic impact [19,24] and blast applications [13,37]. In addition, the topological design of the bioinspired structural characteristics, such as the tablet shape and size, can be tuned for specific applications to improve the interlocking effect of the tablets, which contributes to the toughening and stiffening mechanisms of the nacre-like composites [38]. It is acknowledged that more numerical and experimental work must be performed to fully understand the nacre-like bioinspired composites.

Several structural parameters that may influence the bioinspired composite’s ballistic performance, including the tablets’ waviness and the interfaces’ cohesive properties [4,9], should be further investigated. The results also showed that at lower impact velocities, the nacre-like plate’s ballistic performance was similar to that of the bulk plate, indicating that the ballistic performance improvement over the bulk plate depends on the projectile velocity. The numerical simulations showed that the ballistic performance of the plates also depends on the mechanical response of the projectile. Therefore, further work assessing all the parameters mentioned above should be performed for the optimal design of lightweight bioinspired aluminum alloy composites with improved ballistic performance.

The FE models developed for the ballistic impact simulations in this work could be used to design better experimental testing programs and reduce the number of ballistic tests required in an investigation, which are expensive and time-consuming. The numerical simulations could also be used to assess the ballistic performance of nacre-like composites made of different materials and configurations than those used here. Furthermore, the FE models could be extended to investigate bioinspired nacre-like composites made via 3D printing techniques, which introduce anisotropy [39], porosity [40] and defects [41], which can further influence the ballistic performance.

## 4. Conclusions

In this work, finite element (FE) simulations were performed to evaluate the ballistic performance of nacre-like aluminum alloy 7075-T651 (AA7075) composite plates, composite AA7075 plates with continuous layers (wavy and flat) and bulk monolithic AA7075 plates impacted by rigid and elastoplastic projectiles with impact velocities of 300–600 m/s. It was found that the nacre plate showed improved ballistic performance compared to the bulk plate and the plates with continuous layers. The following conclusions can be drawn from the results of this study:

- The nacre plate exhibited a more ductile failure compared to the brittle failure of the bulk plate.
- The nacre plate exhibited better ballistic performance than the plates with continuous layers, showing that using tablets resulted in a larger area of plastic deformation, producing higher impact energy absorption.
- The structural design of the nacre plate produced an improved ballistic performance by enabling localized energy absorption via the plastic deformation of the tablets and globalized energy dissipation due to interface debonding and friction.
- All the plate configurations showed a better ballistic performance when impacted by an elastoplastic projectile compared to the rigid one, which is explained by the plastic deformation of the elastoplastic projectile and the enlarged contact areas between the projectile and the plates, enabling more energy absorption by the plates.
- The numerical results indicated that the nacre-like composites have the potential to be used for ballistic applications; however, further research is required to assess the parameters that affect the mechanical response of the bioinspired composites to achieve an optimal design with improved ballistic performance.

## Figures and Tables

**Table 1:** Material properties of the aluminum alloy and stainless steel.

Material Properties	AA 7075 [28]	SS 316L
Density ρ (kg/m^3^)	2700	7850
Young’s modulus *E* (GPa)	70	210
Poisson’s ratio ν (-)	0.33	0.3
Inelastic heat fraction η (-)	0.9	0.9
Specific heat *C_p_* (J/kgK)	910	500

**Table 2:** Johnson–Cook plasticity model parameters for the aluminum alloy and stainless steel.

Johnson–Cook Model Parameters	AA 7075 [28]	SS 316L [29]
*A* (MPa)	520	238
*B* (MPa)	477	1202.4
*n* (-)	0.52	0.675
*C* (-)	0.001	0.0224
Reference strain rate ${\dot{\varepsilon }}_{0}$ (s^−1^)	5 × 10^−4^	5 × 10^−4^
*m* (-)	1	1.083
Reference temperature *T_r_* (K)	293	293
Melting temperature *T_m_* (K)	893	1673

**Table 3:** Johnson–Cook damage criterion parameters for the aluminum alloy.

Johnson–Cook Damage Criterion Parameters	AA 7075 [30,31]
$D_{1}$	0.096
$D_{2}$	0.049
$D_{3}$	3.465
$D_{4}$	0.016
$D_{5}$	1.099

**Table 4:** Material properties and cohesive element parameters of the epoxy resin.

Material Properties	Betamate 1044 [33]
Density ρ (kg/m^3^)	1350
Elastic modulus in the normal direction *E* (GPa)	3.1
Elastic modulus in the transverse directions *G*_1_, *G*_2_ (GPa)	1.55
$\mathrm{Maximum}\;\mathrm{normal}\;\mathrm{traction}\;t_{n}$ (MPa)	85.5
$\mathrm{Maximum}\;\mathrm{shear}\;\mathrm{tractions}\;t_{s}$$,\;t_{t}$ (MPa)	70
Critical fracture energy in mode I *G_IC_* (J/m^2^)	1680
Critical fracture energy in mode II *G_IIC_* (J/m^2^)	3570

## Data Availability

The data presented in this study are available on request from the corresponding author.

## References

[1] Zaera R., Abrate S. (2011). Ballistic Impacts on Polymer Matrix Composites, Composite Armor, Personal Armor. Impact Engineering of Composite Structures.

[2] Wegst U.G.K., Bai H., Saiz E., Tomsia A.P., Ritchie R.O. (2015). Bioinspired structural materials. Nat. Mater..

[3] Chen P.-Y., McKittrick J., Meyers M.A. (2012). Biological materials: Functional adaptations and bioinspired designs. Prog. Mater. Sci..

[4] Espinosa H.D., Rim J.E., Barthelat F., Buehler M.J. (2009). Merger of structure and material in nacre and bone—Perspectives on de novo biomimetic materials. Prog. Mater. Sci..

[5] Kakisawa H., Sumitomo T. (2011). The toughening mechanism of nacre and structural materials inspired by nacre. Sci. Technol. Adv. Mater..

[6] Tang B., Niu S., Yang J., Shao C., Wang M., Ni J., Zhang X., Yang X. (2022). Investigation of Bioinspired Nacreous Structure on Strength and Toughness. Biomimetics.

[7] Sun J., Bhushan B. (2012). Hierarchical structure and mechanical properties of nacre: A review. RSC Adv..

[8] Zhang X., Wu K., Ni Y., He L. (2022). Anomalous inapplicability of nacre-like architectures as impact-resistant templates in a wide range of impact velocities. Nat. Commun..

[9] Barthelat F., Tang H., Zavattieri P.D., Li C.M., Espinosa H.D. (2007). On the mechanics of mother-of-pearl: A key feature in the material hierarchical structure. J. Mech. Phys. Solids.

[10] Yao H., Song Z., Xu Z., Gao H. (2013). Cracks fail to intensify stress in nacreous composites. Compos. Sci. Technol..

[11] Huang Z., Li H., Pan Z., Wei Q., Chao Y.J., Li X. (2011). Uncovering high-strain rate protection mechanism in nacre. Sci. Rep..

[12] Barthelat F., Espinosa H.D. (2007). An Experimental Investigation of Deformation and Fracture of Nacre–Mother of Pearl. Exp. Mech..

[13] Flores-Johnson E.A., Shen L., Guiamatsia I., Nguyen G.D. (2015). A numerical study of bioinspired nacre-like composite plates under blast loading. Compos. Struct..

[14] Tran P., Ngo T.D., Ghazlan A., Hui D. (2017). Bimaterial 3D printing and numerical analysis of bio-inspired composite structures under in-plane and transverse loadings. Compos. Part B.

[15] Barthelat F., Zhu D. (2011). A novel biomimetic material duplicating the structure and mechanics of natural nacre. J. Mater. Res..

[16] Ko K., Jin S., Lee S.E., Hong J.-W. (2020). Impact resistance of nacre-like composites diversely patterned by 3D printing. Compos. Struct..

[17] Wu K., Zheng Z., Zhang S., He L., Yao H., Gong X., Ni Y. (2019). Interfacial strength-controlled energy dissipation mechanism and optimization in impact-resistant nacreous structure. Mater. Des..

[18] Yin Z., Hannard F., Barthelat F. (2019). Impact-resistant nacre-like transparent materials. Science.

[19] Miao T., Shen L., Xu Q., Flores-Johnson E.A., Zhang J., Lu G. (2019). Ballistic performance of bioinspired nacre-like aluminium composite plates. Compos. Part B.

[20] Knipprath C., Bond I.P., Trask R.S. (2011). Biologically inspired crack delocalization in a high strain-rate environment. J. R. Soc. Interface.

[21] Yang H., Gao D., Chen P., Lu G. (2023). Numerical Investigation on the Ballistic Performance of Semi-Cylindrical Nacre-like Composite Shells under High-Velocity Impact. Materials.

[22] Gao D., Chen P., Lu G., Yang H. (2023). Numerical analysis for impact resistance of nacre-like composites. Mater. Today Commun..

[23] Ghazlan A., Ngo T., Tan P., Tran P., Xie Y.M. (2023). A Numerical Modelling Framework for Investigating the Ballistic Performance of Bio-Inspired Body Armours. Biomimetics.

[24] Flores-Johnson E.A., Shen L., Guiamatsia I., Nguyen G.D. (2014). Numerical investigation of the impact behaviour of bioinspired nacre-like aluminium composite plates. Compos. Sci. Technol..

[25] ABAQUS (2015). Abaqus Analysis User’s Guide, Version 2016.

[26] Johnson G.R., Cook W.H. A constitutive model and data for materials subjected to large strains, high strain rates, and high temperatures. Proceedings of the 7th International Symposium on Ballistics.

[27] Johnson G.R., Cook W.H. (1985). Fracture characteristics of three metals subjected to various strains, strain rates, temperatures and pressures. Eng. Fract. Mech..

[28] Børvik T., Hopperstad O.S., Pedersen K.O. (2010). Quasi-brittle fracture during structural impact of AA7075-T651 aluminium plates. Int. J. Impact Eng..

[29] Flores-Johnson E.A., Muránsky O., Hamelin C.J., Bendeich P.J., Edwards L. (2012). Numerical analysis of the effect of weld-induced residual stress and plastic damage on the ballistic performance of welded steel plate. Comput. Mater. Sci..

[30] Dorogoy A., Karp B., Rittel D. (2011). A Shear Compression Disk Specimen with Controlled Stress Triaxiality under Quasi-Static Loading. Exp. Mech..

[31] Brar N.S., Joshi V.S., Harris B.W. (2009). Constitutive model constants for Al7075-T651 and Al7075-T6. AIP Conf. Proc..

[32] Benzeggagh M.L., Kenane M. (1996). Measurement of mixed-mode delamination fracture toughness of unidirectional glass/epoxy composites with mixed-mode bending apparatus. Compos. Sci. Technol..

[33] Wang R.X., Shayganpur A., Sareskani S., Spelt J.K. (2006). Analytical peel load prediction as a function of adhesive stress concentration. J. Adhes..

[34] Recht R.F., Ipson T.W. (1963). Ballistic Perforation Dynamics. J. Appl. Mech..

[35] Dey S., Børvik T., Teng X., Wierzbicki T., Hopperstad O.S. (2007). On the ballistic resistance of double-layered steel plates: An experimental and numerical investigation. Int. J. Solids Struct..

[36] Flores-Johnson E.A., Saleh M., Edwards L. (2011). Ballistic performance of multi-layered metallic plates impacted by a 7.62-mm APM2 projectile. Int. J. Impact Eng..

[37] Ghazlan A., Ngo T., Van Le T., Nguyen T., Remennikov A. (2020). Blast performance of a bio-mimetic panel based on the structure of nacre—A numerical study. Compos. Struct..

[38] Zhang Q., Li H., Liu Y., Zhang Z., Yuan Y. (2022). Nacre-inspired topological design tuning the impact resistant behaviors of composite plates. Compos. Struct..

[39] Bahrami B., Mehraban M.R., Koloor S.S.R., Ayatollahi M.R. (2023). Non-local and local criteria based on the extended finite element method (XFEM) for fracture simulation of anisotropic 3D-printed polymeric components. Rapid Prototyp. J..

[40] Khorasani M., Leary M., Downing D., Rogers J., Ghasemi A., Gibson I., Brudler S., Rolfe B., Brandt M., Bateman S. (2023). Numerical and experimental investigations on manufacturability of Al–Si–10Mg thin wall structures made by LB-PBF. Thin-Walled Struct..

[41] Agaliotis E.M., Ake-Concha B.D., May-Pat A., Morales-Arias J.P., Bernal C., Valadez-Gonzalez A., Herrera-Franco P.J., Proust G., Koh-Dzul J.F., Carrillo J.G. (2022). Tensile Behavior of 3D Printed Polylactic Acid (PLA) Based Composites Reinforced with Natural Fiber. Polymers.

